# Multiple trait breeding programs with genotype-by-environment interactions based on reaction norms, with application to genetic improvement of disease resilience

**DOI:** 10.1186/s12711-021-00687-2

**Published:** 2021-12-13

**Authors:** Jack C. M. Dekkers

**Affiliations:** Department of Animal Science, Iowa State University, Ames, USA

## Abstract

**Background:**

Genotype-by-environment interactions for a trait can be modeled using multiple-trait, i.e. character-state, models, that consider the phenotype as a different trait in each environment, or using reaction norm models based on a functional relationship, usually linear, between phenotype and a quantitative measure of the quality of the environment. The equivalence between character-state and reaction norm models has been demonstrated for a single trait. The objectives of this study were to extend the equivalence of the reaction norm and character-state models to a multiple-trait setting and to both genetic and environmental effects, and to illustrate the application of this equivalence to the design and optimization of breeding programs for disease resilience.

**Methods:**

Equivalencies between reaction norm and character-state models for multiple-trait phenotypes were derived at the genetic and environmental levels, which demonstrates how multiple-trait reaction norm parameters can be derived from multiple-trait character state parameters. Methods were applied to optimize selection for a multiple-trait breeding goal in a target environment based on phenotypes collected in a healthy and disease-challenged environment, and to optimize the environment in which disease-challenge phenotypes should be collected.

**Results and conclusions:**

The equivalence between multiple-trait reaction norm and multiple-trait character-state parameters allow genetic improvement for a multiple-trait breeding goal in a target environment to be optimized without recording phenotypes and estimating parameters for the target environment.

## Background

In animal breeding, genotype-by-environment interactions (GxE) have traditionally been modelled by considering that a phenotype recorded in a specific environment is a different genetic trait compared to the same phenotype recorded in a different environment. In these cases, GxE can be quantified based on the genetic correlation between the trait evaluated in different environments [1]. However, if the environmental conditions that affect a phenotype can be quantified in terms of one or several continuous variables, GxE can also be modeled using random regression models [2] by modelling the phenotype as a polynomial function of these continuous variables. In evolutionary biology, the latter are referred to as reaction norm models, while multiple-trait models for GxE are referred to as character-state models [3]. Reaction norm models enable modelling and prediction of breeding values and phenotypes across a range of environments, while predictions for character-state models are limited to the environments in which phenotypes have been evaluated. This enables reaction norm models to be used to optimize breeding programs in terms of the best environment(s) in which to record phenotypes, in order to maximize the rate of genetic improvement in a target environment [4].

Van Tienderen and Koelewijn [5] and de Jong and Bijma [3] demonstrated the mathematical equivalence between the reaction norm model and the character-state model for the specific environments that the character-state model is defined for. This equivalence can be used to derive parameters for a reaction norm model when only estimates of genetic parameters from a character-state model are available, which would be the case when phenotypes are available for only a small number of discrete environments. Alternatively, this equivalence can be used to derive genetic parameters for specific environments when estimates of reaction norm parameters are available, e.g. based on random regression models [2, 6]. For this purpose, linear reaction norms have been used most extensively, with the slope of the phenotypic or breeding value of the trait quantifying the change in the phenotype as a function of the environment, which is referred to as phenotypic plasticity in evolutionary biology [3] and macro-environmental sensitivity [7], robustness, or resilience [8] in animal breeding.

The equivalence between the reaction norm and character state models has been limited to a single trait [3, 5], which is the most relevant case in evolutionary biology, since selection is on the single trait of fitness. However, in animal and plant breeding, selection is typically on multiple traits, which each can have their own reaction norm model. In addition, most reaction norm models only consider a reaction norm for the genetic component of phenotype, although random environmental components of phenotype can also be subject to reaction norms. Thus, the first objective of this study was to extend the equivalence of the reaction norm and character-state models to a multiple-trait setting and to both genetic and environmental effects.

A second objective was to illustrate the application of these methods to the design and optimization of multiple trait nucleus breeding programs that are typical for swine and poultry breeding, in the presence of GxE resulting from disease. In such breeding programs, phenotype recording is typically in the high-health environment of the nucleus. To accommodate GxE, phenotype recording in a commercial-type environment would need to be added, as proposed in so-called combined crossbred-purebred selection programs [9], which, in addition to GxE, also account for potential differences in the genetic basis of traits in crossbreds versus purebreds [10]. For this purpose, choice of the type of commercial environment becomes important and an extreme commercial environment in terms of severity of disease could be considered. An example of the latter would be the natural polymicrobial disease challenge that was described by Putz et al. [11]. Note that the nucleus and disease challenge environments could represent the extremes of the range of commercial environments that are the target of the breeding program. Thus, the second objective of this study was to use multi-trait reaction norm models to optimize selection for a target environment based on phenotype recording in a high-health (i.e. nucleus) and a disease challenge environment, with the target being either a single environment or a range of environments. The third and final objective was to illustrate the use of the methods to optimize the choice of the recording environment.

Related studies have been conducted by Kolmodin and Bijma [4] and Mulder [12]. Kolmodin and Bijma [4] showed that the target environment may not be the optimal recording environment. However, they considered only a single trait and a single recording environment. Mulder [12] considered a situation where both the data recording and the target environments consisted of a normally distributed range of environments. Again, only a single trait was considered. The present study considers two recording environments and either a single or a range of target environments, as well as multiple traits.

## Methods

### Equivalence between character-state and reaction norm models

In a genetic reaction norm model, the impact of the environment on the breeding value of an individual for trait $i$ in environment $j$, ${g}_{ij}$, is modeled as:

1 $${g}_{ij}=\left[\begin{array}{ccc}1& {x}_{1ij}& {x}_{2ij}\dots \end{array} {x}_{mij}\right]{\left[\begin{array}{c}{\gamma }_{0i}\\ \begin{array}{c}{\gamma}_{1i}\\ {\gamma }_{2i}\end{array}\\ \vdots \\ {\gamma }_{mi}\end{array}\right]={\mathbf{x}}_{{\varvec{ij}}}^{\boldsymbol{^{\prime}}}}{{\varvec{\upgamma}}}_{{\varvec{i}}},$$

where, ${x}_{kij}$, for $k=0\dots m$, are covariates that characterize the environment, representing different environmental factors (e.g. temperature, humidity), or the average performance of the contemporary group, and/or their polynomial expansions [5], and ${\gamma }_{ki}$ is the individual’s random regression breeding value associated with covariate ${x}_{kij}$, with variance–covariance structure captured by the $m\times m$ matrix *var*(${{\varvec{\upgamma}}}_{{\varvec{i}}}$). Note that ${\gamma }_{0i}$ is the individual’s intercept breeding value for trait $i$, i.e. for an environment with environmental covariates equal to 0. A similar reaction norm model can be applied to each term in the standard quantitative genetic model for an individual’s phenotype for trait $i$ in environment $j$:

$${y}_{ij}={m}_{ij}+{g}_{ij}+{e}_{ij},$$

where ${m}_{ij}$ is the mean phenotype for trait $i$ in environment $j$ and ${e}_{ij}$ is the individual’s random environmental effect for trait $i$ in environment $j$:

for the mean:

2 $${{m}_{ij}}=\left[\begin{array}{ccc}1& {x}_{1ij}& {x}_{2ij}\dots \end{array} {x}_{mij}\right]{\left[\begin{array}{c}{\mu }_{0i}\\ \begin{array}{c}{\mu }_{1i}\\ {\mu }_{2i}\end{array}\\ \vdots \\ {\mu }_{mi}\end{array}\right]={\mathbf{x}}_{{\varvec{ij}}}^{\boldsymbol{^{\prime}}}}{{\varvec{\upmu}}}_{{\varvec{i}}},$$

where ${\mu }_{ki}$ are fixed regression coefficients associated with covariate ${x}_{kij}$;

for the random environmental effect:

3 $${e}_{ij}=\left[\begin{array}{ccc}1& {x}_{1ij}& {x}_{2ij}\end{array}\dots {x}_{mij}\right]{\left[\begin{array}{c}{\varepsilon }_{0i}\\ \begin{array}{c}{\varepsilon }_{1i}\\ {\varepsilon }_{2i}\end{array}\\ \vdots \end{array}\right]={\mathbf{x}}_{{\varvec{ij}}}^{\boldsymbol{^{\prime}}}}{{\varvec{\upvarepsilon}}}_{{\varvec{i}}},$$

where ${\varepsilon }_{ki}$ are random environmental regression coefficients associated with covariate ${x}_{kij}$, with variance–covariance structure captured by the $m\times m$ matrix $var({{\varvec{\upvarepsilon}}}_{{\varvec{i}}}$). Note that the reaction norm covariates for the mean, the breeding value, and the random environmental effects do not have to be the same.

Breeding values of an individual for trait $i$ in $J$ environments, each considered as a different trait in the character-state model, can be represented by a multi-environment reaction norm model as follows:

4 $${\mathbf{g}}_{{\varvec{i}}}=\left[\begin{array}{c}{g}_{i1}\\ {g}_{i2}\\ \begin{array}{c}\begin{array}{c}{g}_{i3}\\ .\end{array}\\ \begin{array}{c}.\\ {g}_{iJ}\end{array}\end{array}\end{array}\right]=\left[\begin{array}{ccccc}1& {{x}_{1i1}}& {{x}_{2i1}}& \cdots & {{x}_{mi1}}\\ 1& {{x}_{1i2}}& {{x}_{2i2}}& \cdots & {{x}_{mi2}}\\ 1& {{x}_{1i3}}& {{x}_{2i3}}& \cdots & {{x}_{mi3}}\\ \cdots & \cdots & \cdots & \cdots & \cdots \\ 1& {{x}_{1iJ}}& {{x}_{2iJ}}& \cdots & {{x}_{miJ}}\end{array}\right]{\left[\begin{array}{c}{\gamma }_{0i}\\ {\gamma }_{1i}\\ \begin{array}{c}\begin{array}{c}{\gamma }_{2i}\\ .\end{array}\\ \begin{array}{c}.\\ {\gamma }_{mi}\end{array}\end{array}\end{array}\right]={\mathbf{X}}_{{\varvec{i}}}^{\boldsymbol{^{\prime}}}}{{\varvec{\upgamma}}}_{{\varvec{i}}}.$$

This results in the following equivalence between the variance–covariance structure of multi-environment breeding values in the character-state model and the variance–covariance structure of breeding values in the reaction norm model:

5 $$var\left({\mathbf{g}}_{i}\right)={\mathbf{X}}_{i}^{\boldsymbol{^{\prime}}}var({{\varvec{\upgamma}}}_{i}){\mathbf{X}}_{i}.$$

This is the single-trait relationship between character-state and reaction model parameters derived previously [5]. A similar relationship holds for the random environmental effects:

6 $$var\left({\mathbf{e}}_{{\varvec{i}}}\right)={\mathbf{X}}_{{\varvec{i}}}^{\boldsymbol{^{\prime}}}var\left({{\varvec{\upvarepsilon}}}_{{\varvec{i}}}\right){\mathbf{X}}_{{\varvec{i}}}.$$

These single-trait relationships can be expanded to $I$ traits in $J$ environments, for a total of $I\times J$ character-states and $I\times m$ reaction norm variables. For the vector of breeding values for $I$ traits in $J$ environments, we get:

7 $$\mathbf{g}=\left[\begin{array}{c}{\mathbf{g_{1}}}\\ {\mathbf{g_{2}}}\\ \begin{array}{c}\begin{array}{c}\vdots \\ .\end{array}\\ \begin{array}{c}.\\ {\mathbf{g}}_{{\varvec{I}}}\end{array}\end{array}\end{array}\right]=\left[\begin{array}{c}{g}_{11}\\ {g}_{12}\\ \begin{array}{c}\begin{array}{c}\vdots \\ \begin{array}{c}{g}_{1J}\\ {g}_{21}\end{array}\end{array}\\ \begin{array}{c}{g}_{22}\\ \vdots \\ \begin{array}{c}{g}_{2J}\\ \vdots \\ \begin{array}{c}{g}_{I1}\\ {g}_{I2}\\ \begin{array}{c}\vdots \\ {g}_{\mathrm{IJ}}\end{array}\end{array}\end{array}\end{array}\end{array}\end{array}\right]=\left[\begin{array}{ccccc}{\mathbf{X_{1}}}^{\boldsymbol{^{\prime}}}& \bf{0}& \bf{0}& \cdots & \bf{0}\\ \bf{0}& {\mathbf{X_{2}}}^{\boldsymbol{^{\prime}}}& \bf{0}& \cdots & \bf{0}\\ \bf{0}& \bf{0}& {\mathbf{X_{3}}}^{\boldsymbol{^{\prime}}}& \cdots & \bf{0}\\ \cdots &\cdots & \cdots & \cdots & \cdots \\ \bf{0}& \bf{0}& \bf{0}& \cdots & {\mathbf{X}}_{{\varvec{I}}}^{\boldsymbol{^{\prime}}}\end{array}\right]\left[\begin{array}{c}{{\varvec{\upgamma}}}_{1}\\ {{\varvec{\upgamma}}}_{2}\\ \begin{array}{c}\begin{array}{c}\vdots \\ .\end{array}\\ \begin{array}{c}.\\ {{\varvec{\upgamma}}}_{{\varvec{I}}}\end{array}\end{array}\end{array}\right]={\mathbf{X}}^{\boldsymbol{^{\prime}}}{\varvec{\upgamma}},$$

with the following relationship between the $(I\times J)\times (I\times J)$ variance–covariance matrix for the character-state model and the $(I\times m)\times (I\times m)$ variance–covariance matrix for the reaction norm model:

8 $$var\left(\mathbf{g}\right)={\mathbf{X}}^{\boldsymbol{^{\prime}}}var\left({\varvec{\upgamma}}\right)\mathbf{X}$$

with

9 $$var\left({\varvec{\upgamma}}\right)=\left[\begin{array}{ccccc}var\left({{\varvec{\upgamma}}}_{\bf{1}}\right)& cov\left({{\varvec{\upgamma}}}_{\bf{1}},{{\varvec{\upgamma}}}_{\bf{2}}\right)& cov\left({{\varvec{\upgamma}}}_{\bf{1}},{{\varvec{\upgamma}}}_{\bf{3}}\right)& \cdots & cov\left({{\varvec{\upgamma}}}_{\bf{1}},{{\varvec{\upgamma}}}_{{\varvec{I}}}\right)\\ cov\left({{\varvec{\upgamma}}}_{\bf{2}},{{\varvec{\upgamma}}}_{\bf{1}}\right)& var\left({{\varvec{\upgamma}}}_{\bf{2}}\right)& cov\left({{\varvec{\upgamma}}}_{\bf{2}},{{\varvec{\upgamma}}}_{\bf{3}}\right)& \cdots & cov\left({{\varvec{\upgamma}}}_{\bf{2}},{{\varvec{\upgamma}}}_{{\varvec{I}}}\right)\\ cov\left({{\varvec{\upgamma}}}_{\bf{3}},{{\varvec{\upgamma}}}_{\bf{1}}\right)& cov\left({{\varvec{\upgamma}}}_{\bf{1}},{{\varvec{\upgamma}}}_{\bf{2}}\right)& var\left({{\varvec{\upgamma}}}_{\bf{3}}\right)& \cdots & cov\left({{\varvec{\upgamma}}}_{\bf{3}},{{\varvec{\upgamma}}}_{{\varvec{I}}}\right)\\ \cdots & \cdots & \cdots & \cdots & \cdots \\ cov\left({{\varvec{\upgamma}}}_{{\varvec{I}}},{{\varvec{\upgamma}}}_{\bf{1}}\right)& cov\left({{\varvec{\upgamma}}}_{{\varvec{I}}},{{\varvec{\upgamma}}}_{\bf{2}}\right)& cov\left({{\varvec{\upgamma}}}_{{\varvec{I}}},{{\varvec{\upgamma}}}_{\bf{3}}\right)& \cdots & var\left({{\varvec{\upgamma}}}_{{\varvec{I}}}\right)\end{array}\right].$$

Similarly, for the random environmental effects for $I$ traits in $J$ environments:

10 $$\mathbf{e}=\left[\begin{array}{c}{\mathbf{e_{1}}}\\ {\mathbf{e_{2}}}\\ \begin{array}{c}\begin{array}{c}\vdots \\ .\end{array}\\ \begin{array}{c}.\\ {\mathbf{e}}_{\mathbf{I}}\end{array}\end{array}\end{array}\right]=\left[\begin{array}{c}{e}_{11}\\ {e}_{12}\\ \begin{array}{c}\begin{array}{c}\vdots \\ \begin{array}{c}{e}_{1J}\\ {e}_{21}\end{array}\end{array}\\ \begin{array}{c}{e}_{22}\\ \vdots \\ \begin{array}{c}{e}_{2J}\\ \vdots \\ \begin{array}{c}{e}_{I1}\\ {e}_{I2}\\ \begin{array}{c}\vdots \\ {e}_{IJ}\end{array}\end{array}\end{array}\end{array}\end{array}\end{array}\right]=\left[\begin{array}{ccccc}{\mathbf{X_{1}}}^{\boldsymbol{^{\prime}}}& \bf{0}& \bf{0}& \cdots & \bf{0}\\ \bf{0}& {\mathbf{X_{2}}}^{\boldsymbol{^{\prime}}}& \bf{0}& \cdots & \bf{0}\\ \bf{0}& \bf{0}& {\mathbf{X_{3}}}^{\boldsymbol{^{\prime}}}& \cdots & \bf{0}\\ \cdots & \cdots & \cdots & \cdots & \cdots \\ \bf{0}& \bf{0}& \bf{0}& \cdots & {\mathbf{X}}_{{\varvec{I}}}^{\boldsymbol{^{\prime}}}\end{array}\right]\left[\begin{array}{c}{{\varvec{\upvarepsilon}}}_{\bf{1}}\\ {{\varvec{\upvarepsilon}}}_{\bf{2}}\\ \begin{array}{c}\begin{array}{c}\vdots \\ .\end{array}\\ \begin{array}{c}.\\ {{\varvec{\upvarepsilon}}}_{\mathbf{I}}\end{array}\end{array}\end{array}\right]={\mathbf{X}}^{\boldsymbol{^{\prime}}}{\varvec{\upvarepsilon}},$$

and

11 $$var\left(\mathbf{e}\right)={\mathbf{X}}^{\boldsymbol{^{\prime}}}var\left({\varvec{\upvarepsilon}}\right)\mathbf{X},$$

with

12 $$var\left({\varvec{\upvarepsilon}}\right)=\left[\begin{array}{ccccc}var\left({{\varvec{\upvarepsilon}}}_{\bf{1}}\right)& cov\left({{\varvec{\upvarepsilon}}}_{\bf{1}},{{\varvec{\upvarepsilon}}}_{\bf{2}}\right)& cov\left({{\varvec{\upvarepsilon}}}_{\bf{1}},{{\varvec{\upvarepsilon}}}_{\bf{3}}\right)& \cdots & cov\left({{\varvec{\upvarepsilon}}}_{\bf{1}},{{\varvec{\upvarepsilon}}}_{{\varvec{I}}}\right)\\ cov\left({{\varvec{\upvarepsilon}}}_{\bf{2}},{{\varvec{\upvarepsilon}}}_{\bf{1}}\right)& var\left({{\varvec{\upvarepsilon}}}_{\bf{2}}\right)& cov\left({{\varvec{\upvarepsilon}}}_{\bf{2}},{{\varvec{\upvarepsilon}}}_{\bf{3}}\right)& \cdots & cov\left({{\varvec{\upvarepsilon}}}_{\bf{2}},{{\varvec{\upvarepsilon}}}_{{\varvec{I}}}\right)\\ cov\left({{\varvec{\upvarepsilon}}}_{\bf{3}},{{\varvec{\upvarepsilon}}}_{\bf{1}}\right)& cov\left({{\varvec{\upvarepsilon}}}_{\bf{1}},{{\varvec{\upvarepsilon}}}_{\bf{2}}\right)& var\left({{\varvec{\upvarepsilon}}}_{\bf{3}}\right)& \cdots & cov\left({{\varvec{\upvarepsilon}}}_{\bf{3}},{{\varvec{\upvarepsilon}}}_{{\varvec{I}}}\right)\\ \cdots & \cdots & \cdots & \cdots & \cdots \\ cov\left({{\varvec{\upvarepsilon}}}_{{\varvec{I}}},{{\varvec{\upvarepsilon}}}_{\bf{1}}\right)& cov\left({{\varvec{\upvarepsilon}}}_{{\varvec{I}}},{{\varvec{\upvarepsilon}}}_{\bf{2}}\right)& cov\left({{\varvec{\upvarepsilon}}}_{{\varvec{I}}},{{\varvec{\upvarepsilon}}}_{\bf{3}}\right)& \cdots & var\left({{\varvec{\upvarepsilon}}}_{{\varvec{I}}}\right)\end{array}\right].$$

### Optimizing genetic improvement for a target environment using linear reaction norms

The above equivalences between character-state and reaction norm parameters can be used to design and optimize genetic improvement for a target environment. For illustration, a case is considered here where genetic selection occurs in a nucleus herd but phenotypes are recorded in both the high-health nucleus ($N$) and in a chosen ‘challenge’ ($C$) environment. The objective is to maximize genetic improvement for a target environment ($T$) that may be different from the nucleus and the challenge environments. As input, character-state parameters (heritabilities, phenotypic standard deviations, and genetic and phenotypic correlations) are available for phenotypes observed in the nucleus and challenge environments, considering each phenotype to be a different trait in the two environments. To convert these to reaction norm parameters, consider the following linear reaction norm model for phenotype for trait $i$ in environment $j$:

$${y}_{ij}={\mu }_{i}+{\beta }_{j}{x}_{ij}+{\gamma }_{0i}+{\gamma }_{1i}{x}_{ij}+{\varepsilon }_{0i}+{\varepsilon }_{1i}{x}_{ij},$$

where ${\mu }_{i}$ is the mean for trait $i$, ${\beta }_{i}$ is the fixed slope for trait $i$, ${x}_{ij}$ is the environmental covariate for trait $i$ in environment $j$, ${\gamma }_{0i}$ is the breeding value for the intercept for trait $i$, ${\gamma }_{1i}$ is the breeding value for the slope for trait $i$, ${\varepsilon }_{0i}$ is the residual for trait $i$, and ${\varepsilon }_{1i}$ is the slope residual for trait $i$.

Without loss of generality, the environmental covariates can be scaled such that they are 0 in the nucleus (${x}_{iN}$ = 0) and 1 in the challenge environment (${x}_{iC}$ = 1). Then, the model for phenotype for trait $i$ in the nucleus is:

$${y}_{iN}={\mu }_{i}+{\gamma }_{0i}+{\varepsilon }_{0i},$$

and the model for phenotype for trait $j$ in the challenge environment is:

$${y}_{iC}={\mu }_{i}+{\beta }_{j}+{\gamma }_{0i}+{\gamma }_{1i}+{\varepsilon }_{0i}+{\varepsilon }_{1i}.$$

Thus, the model for the breeding value for trait $i$ in the nucleus is: ${g}_{iN}={\gamma }_{0i}$, and the model for the breeding value for trait $i$ in the challenge environment is: ${g}_{iC}={\gamma }_{0i}+{\gamma }_{1i}$.

Thus, the breeding value for the slope of the reaction norm for trait $i$ is: ${\gamma }_{1i}={g}_{iC}-{g}_{iN}$.

Then, the model for the breeding value for trait $i$ in the target environment can be written as:

$${g}_{iT}={\gamma }_{0i}+{\gamma }_{1i}{x}_{iT}.$$

Substituting ${\gamma }_{1i}={g}_{iC}-{g}_{iN}$ results in

13 $${g}_{iT}=\left({1-x}_{iT}\right){g}_{iN}+{x}_{iT}{g}_{iC}.$$

Thus, selecting for ${g}_{iT}$ is equivalent to selecting for $\left({1-x}_{iT}\right){g}_{iN}+{x}_{iT}{g}_{iC}$. This relationship can be used to convert a breeding goal based on traits defined for the target environment into a breeding goal based on traits in the nucleus and challenge environments. I.e., the term ${v}_{iT}{g}_{iT}$ in the breeding goal for the target environment, where ${v}_{iT}$ is the economic value of trait $i$ in the target environment, can be replaced by $\left({1-x}_{iT}\right){v}_{iT}{g}_{iN}+{x}_{iT}{{v}_{iT}g}_{iC}$. Thus, a breeding goal with an economic value of ${v}_{iT}$ for ${g}_{iT}$ is equivalent to a breeding goal based on ${g}_{iN}$ and ${g}_{iC}$, with economic values equal to ${v}_{iT}\left({1-x}_{iT}\right)$ and ${{v}_{iT}x}_{iT}$, respectively. Thus, effectively, the economic values for the trait observed in the nucleus and the challenge environments are weighted by the relative value of the environmental covariate for that trait in the target environment.

Following the same arguments, a multi-trait breeding goal based on $I$ traits in the target environment can be written as:

14 $${{H}_{T}}={\mathbf{v}}_{{\varvec{T}}}^{\boldsymbol{^{\prime}}}{\mathbf{g}}_{{\varvec{T}}}=[\left(\bf{1}-{\mathbf{x}}_{{\varvec{T}}}\right){\circ \mathbf{v}}_{{\varvec{T}}}{]\boldsymbol{^{\prime}}\mathbf{g}}_{{\varvec{N}}}+{[{\mathbf{x}}_{{\varvec{T}}}\circ \mathbf{v}}_{{\varvec{T}}}]\boldsymbol{^{\prime}}{\mathbf{g}}_{{\varvec{C}}},$$

where ${\mathbf{g}}_{{\varvec{j}}}$ is the vector of breeding values for the $I$ traits in environment $j$ ($j=T,N, \mathrm{or}\, C$), ${\mathbf{x}}_{{\varvec{T}}}$ is a vector of environmental covariates, with elements ${x}_{iT}$ for each trait $i$ in environment $T$, ${\mathbf{v}}_{{\varvec{T}}}$ is the vector of economic values, with elements ${v}_{iT}$ for trait $i$ in the target environment, **1** is a vector of 1s, and $\circ$ is the Hadamard (element-wise) product.

Using these same relationships, with selection on phenotypes recorded in the nucleus and challenge environments, genetic change for trait $i$ in the target environment, $\Delta {g}_{iT}$, can also be derived from genetic change for trait $i$ in the nucleus, $\Delta {g}_{iN}$, and in the challenge environment, $\Delta {g}_{iC}$, as:

15 $$\Delta {g}_{iT}=\left({1-x}_{iT}\right){\Delta g}_{iN}+{x}_{iT}{\Delta g}_{iC}.$$

Thus, with phenotypes recorded in the nucleus and challenge environments but not in the target environment, selection of nucleus individuals to maximize response for the breeding goal in the target environment can be derived based on a breeding program that is defined on the basis of genetic traits in the nucleus and challenge environments. Standard selection index theory, as implemented in the SelAction software [13], can be used for this purpose. Note that this requires estimates of character-state genetic parameters among and between all traits observed in the nucleus and the challenge environment but does not require explicit estimates of genetic parameters in the target environment, as these are implicit to the assumed linear reaction norm model.

This approach can also be extended to a target that encompasses a range of environments. For example, if the market that the breeding program targets includes $J$ environments, with each environment having a proportional relevance to the breeding program denoted by ${f}_{J}$ for $j=1,\dots ,J$, environmental covariates ${x}_{ij}$_,_ and economic values ${v}_{ij}$, then the breeding goal to target for selection in the nucleus is:

16 $${H}_{T}=[\mathbf{f}\circ \left(\bf{1}-\mathbf{X}\right)\circ \mathbf{V}]\mathrm{^{\prime}}{\mathbf{g}}_{{\varvec{N}}}+[\mathbf{f}\circ \mathbf{X}\circ \mathbf{V}]\mathrm{^{\prime}}{\mathbf{g}}_{{\varvec{C}}},$$

where $\mathbf{f}$ is a vector of size $J$, ${\mathbf{f}}^{\boldsymbol{^{\prime}}}=[{f}_{1},{f}_{2},\dots , {f}_{J}]$, $\mathbf{V}$ is an $I\times J$ matrix with economic values for each environment $j$, $\mathbf{V}=[{\mathbf{v}}_{\bf{1}},{\mathbf{v}}_{\bf{2}}, \dots , {\mathbf{v}}_{{\varvec{J}}}]$, $\mathbf{X}$ is an $I\times J$ matrix with environmental covariates for each environment $k$, $\mathbf{X}=[{\mathbf{x}}_{\bf{1}},{\mathbf{x}}_{\bf{2}},\dots , {\mathbf{x}}_{{\varvec{J}}}]$, and **1** is a $I\times J$ matrix of 1s.

### Example

To illustrate the method, consider a sire line purebred nucleus breeding program in pigs, with selection for a breeding goal consisting of growth rate and mortality. Both traits are recorded in the high-health nucleus and in a challenge facility, where (crossbred) paternal half-sibs of purebred selection candidates are subjected to a polymicrobial natural disease challenge, e.g. as described by [11] and [14]. Phenotypes recorded in the nucleus and challenge facility are considered separate but correlated traits. Assumed trait parameters are in Table 1. Although mortality is recorded as a binary trait, it is treated as a continuous variable here for simplicity and illustration.

**Table 1:** Trait parameters for average daily gain (ADG, kg/day) and mortality rate (MORT, %) in the nucleus (*N*), challenge (*C*), and target (*T*) environments

Trait	Phenotypic (below diagonal) and genetic (above diagonal) correlations, and heritabilities (on diagonal)						Trait mean	Phenotypic SD	Economic value ($/unit)
	ADG_N_	MORT_*N*_	ADG_*C*_	MORT_*C*_	ADG_*T*_	MORT_*T*_			
ADG_*N*_	0.25	− 0.20	0.60	− 0.20	0.864	− 0.225	0.90	0.135	181
MORT_*N*_	− 0.13	0.03	–	0.40	− 0.221	0.637	5	21.8	− 1
ADG_*C*_	–	− 0.20	0.25	− 0.40	0.921	− 0.395	0.75	0.175	262
MORT_*C*_	–	–	− 0.25	0.07	− 0.347	0.961	20	40.0	− 1
ADG_*T*_	–	–	–	–	0.25	− 0.358	0.825	0.175	219
MORT_*T*_	–	–	–	–	–	0.057	12.5	40.0	− 1

Parameters in the target environment were derived using the reaction norm model

–: not observed

The economic value for growth rate (average daily gain, ADG) was derived based on the following simple cost function for raising grow-finish pigs from 15 kg to the 120 kg market weight: profit/pig = − 1.42(120–15)/ADG, where 1.42 is the cost per day per pig, including housing and feed. Taking the first derivative of this function results in the following economic value for growth rate as a function of the mean growth rate in the given environment, $${\upmu }_{\mathrm{ADG}}:{\mathrm{v}}_{\mathrm{ADG}}=$$1.49/$${\upmu }_{\mathrm{ADG}}^{2}$$. Note that in this example, the economic value of growth rate depends on the trait mean and, therefore, differs as a function of the environmental covariate. The economic value for mortality was assumed to be independent of the population mean and set equal to − $1 per pig per percent increase in mortality rate.

For the breeding program in the closed nucleus, each discrete generation, 75 boars are each mated to 10 sows, for two litters per sow, each litter producing five male and five female selection candidates. Thus, proportions selected are 1/(10 * 10) = 0.01 for males and 1/10 = 0.1 for females. To produce progeny for the challenge facility, the 75 boars are also each mated to ten maternal sows, for two litters each, producing ten progeny per litter, for 200 progeny per boar. The program SelAction [13] was used to predict asymptotic rates of genetic gain from selection in the nucleus for the breeding goal in the target environment, and when phenotypes on growth rate and mortality were recorded either in the nucleus alone or also in the challenge facility. Selection of nucleus males and females was on an index of multiple-trait pseudo best linear unbiased predictions of estimated breeding values (BLUP EBV) [15] derived using own phenotypes, the average phenotypes of nine full sibs and of 190 paternal half-sibs, and the BLUP EBV of the sire, dam, and mates of the sire for growth rate and mortality in the nucleus. With availability of challenge data, the average performances in the challenge facility of 200 paternal half-sibs for growth rate and mortality in the challenge environment were added, as well as the BLUP EBV of the sire, dam, and mates of the sire for these traits.

## Results

### Derivation of genetic parameters for the target environment

For the above example, linear reaction norm models were assumed for both the genetic and environmental effects on phenotype. The environmental covariates (i.e. challenge load for growth rate and mortality) were scaled to be 0 in the nucleus and 1 in the challenge environment, resulting in the following covariate matrix for both the genetic and environmental reaction norm models for growth rate and mortality in the nucleus and challenge environments, with the order of traits and environments as in Table 1: $$\mathbf{X}=\left[\begin{array}{cccc}\bf{1}& \bf{0}& \bf{0}& \bf{0}\\ \bf{0}& \bf{1}& \bf{0}& \bf{0}\\ \bf{1}& \bf{0}& \bf{1}& \bf{0}\\ \bf{0}& \bf{1}& \bf{0}& \bf{1}\end{array}\right]$$.

This matrix can be used to translate the genetic and environmental variance–covariance matrices for the intercept and slope variables of the reaction norm model ($var({\varvec{\upgamma}})$ and $var({\varvec{\upvarepsilon}})$) to those of the character-state model ($var(\mathbf{g})$ and $var(\mathbf{e})$) based on Eqs. (8) and (11) as: $var(\mathbf{g})=\mathbf{X}var({\varvec{\upgamma}})\mathbf{X}{\boldsymbol{^{\prime}}}$ and $var(\mathbf{e})=\mathbf{X}var({\varvec{\upvarepsilon}})\mathbf{X}{\boldsymbol{^{\prime}}}$. Note that the latter requires values for all elements of $var(\mathbf{e})$, some of which are not observable (Table 1) because an animal can only be evaluated in one environment. Here, for simplicity, the unobserved environmental correlations were set equal to the genetic correlations. Note that, when an individual can be phenotyped in only one environment, only environmental correlations between phenotypes recorded in the same environment matter. In the example, variance–covariance matrices at the character-state level are available for the nucleus and challenge environments. These can be converted to variance–covariance matrices at the reaction norm level by inverting Eqs. (8) and (11) as:

$$var\left({\varvec{\upgamma}}\right)={\mathbf{X}}^{-1}var\left(\mathbf{g}\right){{\mathbf{X}}^{\boldsymbol{^{\prime}}}}^{\bf{-1}}=\left[\begin{array}{cccc}0.0046& -0.0510& 0.0035& -0.1400\\ -0.0510& 14.3& -0.0647& 16.0\\ 0.0035& -0.0647& 0.0077& -0.3704\\ -0.1400& 16.0& -0.3704& 112.0\end{array}\right],$$

$$var\left({\varvec{\upvarepsilon}}\right)={\mathbf{X}}^{-1}var\left(\mathbf{e}\right){{\mathbf{X}}^{\boldsymbol{^{\prime}}}}^{\bf{-1}}=\left[\begin{array}{cccc}0.0137& -0.3168& 0.0106& -0.8838\\ -0.3168& 460.8& -0.8838& 331.2\\ 0.0106& -0.8838& 0.0230& -1.3796\\ -0.8838& 331.2& -1.3796& 1488.0\end{array}\right].$$

Based on the reaction norm model parameters, character-state parameters can be derived for any level of the reaction norm covariates. This allows, e.g., genetic parameters and genetic progress in a target environment to be evaluated, with selection on either phenotypes collected in only the nucleus, or phenotypes collected in both the nucleus and the challenge environments. For example, for a target environment that is intermediate to the nucleus and challenge environments for both growth rate and mortality, the genetic variance–covariance matrix can be derived from the genetic variance–covariance matrix of the reaction norm model, using the following reaction norm covariate matrix: $${\mathbf{X}}_{{\varvec{T}}}=\left[\begin{array}{cccc}1& 0& 0& 0\\ 0& 1& 0& 0\\ 1& 0& 1& 0\\ 0& 1& 0& 1\\ 1& 0& 0.5& 0\\ 0& 1& 0& 0.5\end{array}\right]$$, which is equal to $\mathbf{X}$ augmented with a row for ${\mathrm{ADG}}_{T}$ and ${\mathrm{MORT}}_{T}$. This results in the following genetic variance–covariance matrix for growth rate and mortality in the nucleus, challenge, and target environments:

$$var\left({\mathbf{g}}_{{\varvec{T}}}\right)={\mathbf{X}}_{{\varvec{T}}}var\left({\varvec{\upgamma}}\right){{\mathbf{X}}^{\boldsymbol{^{\prime}}}}_{{\varvec{T}}}=\left[\begin{array}{cccccc}0.0046& -0.0510& 0.0035& -0.1400& 0.0041& -0.0955\\ -0.0510& 14.3& -0.0647& 16.0& -0.0578& 15.1\\ 0.0035& -0.0647& 0.0077& -0.3704& 0.0056& -0.2176\\ -0.1400& 16.0& -0.3704& 112.0& -0.2552& 64.0\\ 0.0041& -0.0578& 0.0056& -0.2552& 0.0048& -0.1565\\ -0.0955& 15.1& -0.2176& 64.0& -0.1565& 39.6\end{array}\right].$$

**Fig. 1:**
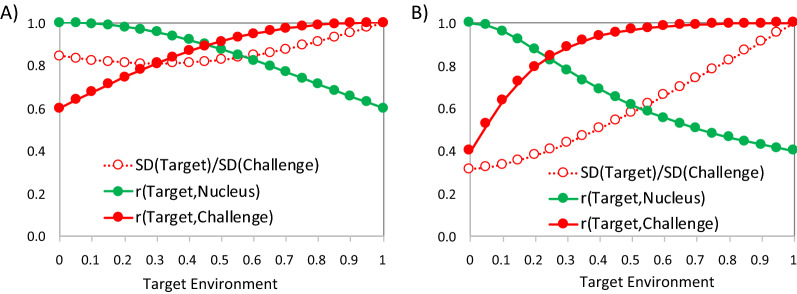
Genetic parameters (genetic correlations, r, of traits in the target with traits in the nucleus and challenge environments, and the ratio of the genetic standard deviation, SD, of traits in the target versus the challenge environment) as a function of the target environment, for **A** average daily gain, and **B** mortality rate. The x-axis represents the reaction norm covariate in the target environment, ranging from the nucleus environment (0) to the challenge environment (1)

Resulting parameters for growth rate and mortality in the target environment are in Table 1. Figure 1 shows how the genetic parameters for growth rate and mortality in the target environment change as a function of the reaction norm covariates for the target environment. In this example the reaction norm covariate in the target environment was assumed to be the same for growth rate and mortality, but this is not necessary. For example, if the target environment is more severe for growth rate than mortality, the last two rows of ${\mathbf{X}}_{{\varvec{T}}}$ could be set to be: $$\left[\begin{array}{cccc}1& 0& 0.6& 0\\ 0& 1& 0& 0.5\end{array}\right]$$.

### Optimizing genetic improvement for the target environment

Based on the equivalence of breeding goals in Eq. (14), genetic gain for a breeding goal defined in a target environment with phenotype recording in the nucleus and challenge environments can either be derived by modeling a 6-trait breeding program (i.e. growth rate and mortality in 3 environments) for the target environment breeding goal ${H}_{T}={\mathbf{v}}_{{\varvec{T}}}^{\boldsymbol{^{\prime}}}{\mathbf{g}}_{{\varvec{T}}}$, using the associated genetic parameters, as derived above from the reaction norm model, or by modelling a 4-trait breeding program (i.e. growth rate and mortality in the nucleus and challenge environments) for the following breeding goal: ${H}_{T}=[\left(\bf{1}-{\mathbf{x}}_{{\varvec{T}}}\right){\circ \mathbf{v}}_{{\varvec{T}}}]\boldsymbol{^{\prime}}{\mathbf{g}}_{{\varvec{N}}}+[{\mathbf{x}}_{{\varvec{T}}}{\circ \mathbf{v}}_{{\varvec{T}}}{]\boldsymbol{^{\prime}}\mathbf{g}}_{{\varvec{C}}}$. In the latter case, genetic gains in the target environment can be derived as a weighted average of genetic gains in the nucleus and challenge environments based on Eq. (15):

$${\Delta \mathbf{g}}_{{\varvec{T}}}=\left(\bf{1}-{\mathbf{x}}_{{\varvec{T}}}\right)\circ {\Delta \mathbf{g}}_{{\varvec{N}}}+{\mathbf{x}}_{{\varvec{T}}}{\circ\Delta \mathbf{g}}_{{\varvec{C}}}.$$

For the example, with the target environment being intermediate to the nucleus and challenge environments for both growth rate and mortality, vectors of economic values for the equivalent 4-trait breeding goal become: $$\left(\left[\begin{array}{c}1\\ 1\end{array}\right]-\left[\begin{array}{c}0.5\\ 0.5\end{array}\right]\right) \circ \left[\begin{array}{c}219\\ -1\end{array}\right]=\left[\begin{array}{c}109.5\\ -0.5\end{array}\right]$$ for ${\mathbf{g}}_{{\varvec{N}}}$ and $$\left[\begin{array}{c}0.5\\ 0.5\end{array}\right]\circ \left[\begin{array}{c}219\\ -1\end{array}\right]=\left[\begin{array}{c}109.5\\ -0.5\end{array}\right]$$ for ${\mathbf{g}}_{{\varvec{C}}}$, and genetic gains for traits in the target environment are $${\Delta \mathbf{g}}_{{\varvec{T}}}={\left(\left[\begin{array}{c}1\\ 1\end{array}\right]-\left[\begin{array}{c}0.5\\ 0.5\end{array}\right]\right)}^{^{\prime}}\circ {\Delta \mathbf{g}}_{{\varvec{N}}}+ {\left[\begin{array}{c}0.5\\ 0.5\end{array}\right]}^{^{\prime}}{\circ\Delta \mathbf{g}}_{{\varvec{C}}}$$.

**Fig. 2:**
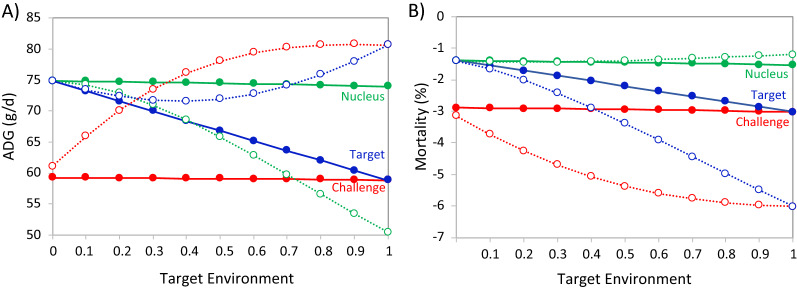
Genetic gain in **A** average daily gain (ADG) and **B** mortality in the nucleus (green), challenge (red), and target (blue) environments when the objective is to maximize the breeding goal in the target environment, without (solid lines) and with (broken lines) recording phenotypes in the challenge environment. The x-axis represents the reaction norm covariate in the target environment, ranging from the nucleus environment (0) to the challenge environment (1)

Figure 2 shows the rate of genetic gain for growth rate and mortality in the nucleus, challenge, and target environments for different target environments, when the objective is to maximize the rate of genetic improvement for the breeding goal in the target environment, both without and with phenotypes recorded in the challenge environment. Each result was based on running SelAction for the 4-trait model (growth rate and mortality in the nucleus and challenge environments), with economic values as derived above for the given reaction norm covariates for the target environment, and genetic gain in the target environment derived as a weighted average of genetic gains in the nucleus and challenge environment based on Eq. (15).

**Fig. 3:**
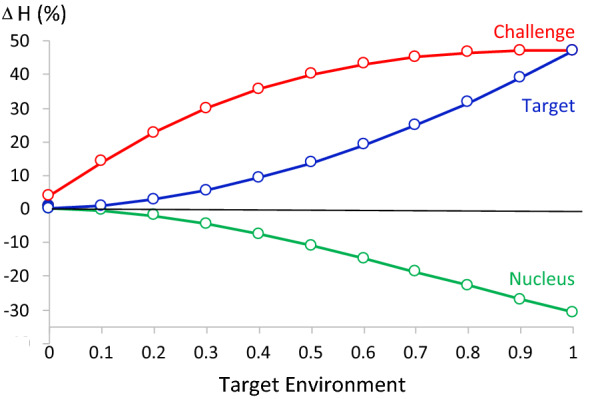
Percentage increase in genetic gain in the breeding goal in the nucleus (green), challenge (red), and target (blue) environments from adding phenotype recording in the challenge environment when the target environment is the breeding objective. The x-axis represents the reaction norm covariate in the target environment, ranging from the nucleus environment (0) to the challenge environment (1)

Figure 3 shows the impact of adding phenotype recording in the challenge environment on response in the breeding goal. Adding data from the challenge environment had little benefit when the nucleus was the target environment because of the low genetic correlations between traits in the challenge and the nucleus environment but increased as the target environment moved closer to the challenge environment. When the challenge environment was the target, gain in the breeding goal increased by over 47% by adding data from the challenge environment. Figure 3 also shows the impact of adding data from the challenge environment on response in the breeding goal at the nucleus and challenge environments, when maximizing gain in the breeding goal defined at the target environment. As expected, adding data from the challenge environment reduced response in the breeding goal at the nucleus when the target environment differed from the nucleus environment, by over 30% when the challenge environment is the target environment.

### Optimizing choice of the recording environment

The developed methods can also be used to determine the optimal environment in which to record phenotypes to maximize genetic improvement for a target environment, with or without availability of phenotypes from the nucleus. To evaluate this scenario, given that genetic parameters are available for traits in the nucleus and the original challenge environment, SelAction can be set up with six traits, i.e. growth rate and mortality in the nucleus, the original challenge environment, and the recording environment, designated with subscript R, with economic values on traits in the nucleus and challenge environment weighted by reaction norm covariates for the target environment, as before, and genetic gain in the target environment derived based on genetic gains in the nucleus and the challenge environment, as before. This does require genetic parameters for the recording environment, which can be derived using a reaction norm covariate matrix ${\mathbf{X}}_{R}$ that is the same as ${\mathbf{X}}_{T}$ but with the last two rows replaced by covariates for the recording environment.

**Fig. 4:**
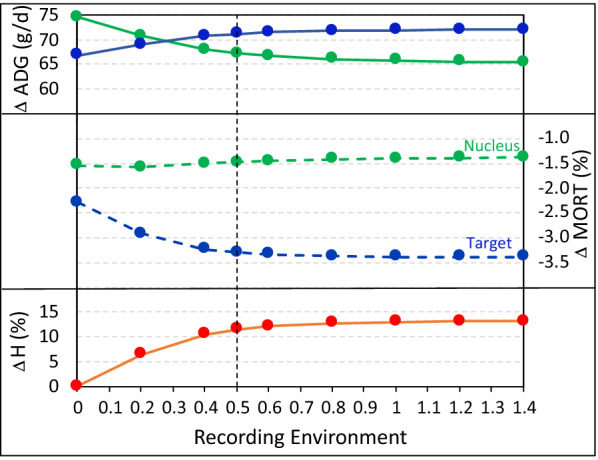
Genetic gain in average daily gain (ΔADG) and mortality (ΔMORT) in the nucleus (green) and target (blue) environments and for the breeding goal (ΔH) in the target environment, as a function of the recording environment. The objective was to maximize the breeding goal in the target environment and the x-axis represents the reaction norm covariate in the recording environment, ranging from the nucleus environment (0) to beyond the original challenge environment (1), with the target environment at 0.5 (vertical broken line). Phenotypes are recorded only in the nucleus and in the recording environment. Genetic gain in the breeding goal for the target environment (ΔH) is expressed as a % above that obtained when the recording environment is the nucleus

Figure 4 shows results of such analyses for recording environments ranging from ${x}_{R}=0$ (i.e. the nucleus environment) to ${x}_{R}=1.4$, i.e. an even more severe challenge than the original challenge environment (${x}_{C}=1$). The target environment was at ${x}_{T}=0.5$, as before. Results show that rates of genetic gain in the target environment increased with increasing severity of the recording environment but at a decreasing rate. Importantly, recording at the target environment did not result in the greatest rates of improvement in the target environment. However, recording under more severe environments than the target environment did not result in substantial additional rates of genetic gain in the target environment. These results are, of course, driven by the specific genetic and economic parameters used here but illustrate the information that can be gained from these analyses to determine the most desirable data recording strategy.

## Discussion

In this study, relationships between character-state and reaction norm models were extended to multiple traits and multiple environments. These relationships enable a smooth transition from character-state to reaction norm models, or vice versa, when modelling GxE. This is important because data may only be available from a limited set of discrete environments, in which case the method enables genetic parameters for unobserved environments to be derived from parameters estimated for the observed environments. Alternatively, data may be available across a continuous range of environments, such that reaction norm parameters can be derived directly, but the breeding program is limited to data recording in only certain environments. In this case, reaction norm parameters can be used to derive character-state parameters for the chosen environments in order to optimize those choices. The example illustrates how the relationship between the reaction norm and character-state models can be used to address a number of questions that are relevant in animal (and plant) breeding. In the following, some issues related to the use of reaction norms for these purposes will be discussed.

### Choice of environmental variables

Key requirements for the use of reaction norm models are that (i) a quantitative measure of the quality of the environment is available and (ii) the functional relationship of that environmental variable with phenotype for the trait of interest is known. Ideally, the environmental factor that drives phenotypic plasticity or GxE is used as the environmental variable [16]. However, the nature of this factor is often not known, let alone its functional relationship with phenotype. In addition, more than one environmental factor may be the driver of phenotypic plasticity [16]. Finally, the drivers of phenotypic plasticity may differ between traits.

In the absence of knowledge of the true drivers of phenotypic plasticity, in practice, a proxy for the quality of the environment is often used in reaction norm models. Fikse et al. [17] investigated the use of alternates of herd-level quantifications of management, genetic composition, and climate to identify the most suitable variables for reaction norm analyses of lactation yield in dairy cattle. Other studies have used estimates of the average phenotype in a given environment as a proxy, as originally proposed by Yates and Cochran [18] and known as Finlay–Wilkinson regression in the plant breeding literature [19]. Gienapp [16] showed that the use of a proxy that is poorly correlated with the true driver of phenotypic plasticity can lead to downward biases in estimates of plasticity or GxE, but that such biases are not present when the environment-specific mean of the trait is used as the proxy, as it implicitly captures all environmental effects on phenotype. Several studies have shown that the best environmental variable can differ between traits and that the best proxy for a given trait may be the environment-specific mean for another trait or for a combination of traits, rather than the mean for the trait itself [20].

When estimating environment-specific means, it is important to remove possible confounding between environmental and genetic effects on phenotype. One solution is to use estimates of contemporary group effects from a mixed linear genetic model for BLUP EBV, as has been applied in many animal breeding applications of reaction norm models [21]. However, Su et al. [22] showed that ignoring the uncertainty of the resulting estimates as a covariate in random regression reaction norm models can result in biased estimates and proposed a one-step hierarchical reaction norm model to overcome this issue.

### Functional relationship between environmental variables and phenotype

In terms of the functional relationship between the environmental variable and phenotype, a simple linear relationship is commonly used in reaction norm models. And, if the environment-specific mean of the trait is used as the environmental variable, a linear relationship with phenotype is expected. However, a linear relationship may not be best for the random genetic and environmental effects, as the aim is to model the variance–covariance structure rather than the phenotypic value of the trait.

Several studies have investigated the use of non-linear reaction norms. Fikse et al. [17] found that quadratic reaction norm covariates of some environmental variables significantly improved the fit of the model for some traits in dairy cattle. Carvalheiro et al. [23] found that quadratic and spline reaction norm models based on estimates of contemporary group effects outperformed the linear reaction norm models for post-weaning weight gain in beef cattle. However, although non-linear reaction norms may provide a better fit to the data, they complicate interpretation and implementation of results. In some cases, a transformation of the environmental variable could simplify the reaction norm to a linear fit.

Non-linear reaction norms are accommodated in the methods developed here; if reaction norm parameters are known or estimated, they can be used to derive parameters for any given set of environments, using Eq. (9). Estimation of non-linear reaction norm parameters can, however, be a challenge, especially across traits.

### Estimation of reaction norm parameters

Random regression models can be used to estimate parameters for reaction norm models if phenotypes are available from multiple quantifiable environments, as described previously. Multiple-trait random regression models (e.g. [24, 25]) would need to be implemented to obtain the parameters required to implement multiple-trait breeding programs. In many applications of random regression models to estimate reaction norm parameters, reaction norms are only implemented at the genetic level and residual variance is either assumed homogeneous or estimated for multiple environmental classes. Meyer [26] and Schnyder et al. [27] showed how heterogenous residual variance could be modelled on a continuous basis using variance functions and fitted in a random regression model.

Alternatively, if variance–covariance estimates from character-state models are available across several discrete environments ($\widehat{var\left(\mathbf{g}\right)}$), these can be used to derive or estimate the variance–covariance matrix for the reaction norm parameters ($\widehat{var\left({\varvec{\upgamma}}\right)}$) using Eq. (9), as was done in the example used here. If the rank of ($\widehat{var\left(\mathbf{g}\right)}$ is equal to the rank of $\mathbf{X}$, then estimates for the reaction norm model can be derived as: $\widehat{var\left({\varvec{\upgamma}}\right)}={\mathbf{X}}^{\boldsymbol{^{\prime}}-1}\widehat{var\left(\mathbf{g}\right)}{\mathbf{X}}^{-1}$. If the chosen rank of $\mathbf{X}$ is smaller than the rank of $\widehat{var\left(\mathbf{g}\right)}$, reduced rank covariance functions [28, 29] can be used to estimate $\widehat{var\left({\varvec{\upgamma}}\right)}$. In addition to reducing complexity, a reduced rank covariance function may provide more accurate estimates because it smooths out estimation errors that are inherent to $\widehat{var\left(\mathbf{g}\right)}$ [29]. Estimates for the reaction norm model cannot be derived from estimates of a character-state model if the rank of $\widehat{var\left(\mathbf{g}\right)}$ is less than the rank of $\mathbf{X}$.

Estimates of the variance–covariance structure of random environmental reaction norms can be obtained in a similar manner from character-state model estimates. However, in character-state models, environmental covariances between environments are often not estimable because an individual may only obtain a phenotype in one environment. For a single trait observed in multiple environments, variance functions could be fitted to estimates of the residual variance in each environment, as described by Meyer [26]. For multiple-trait breeding programs, however, co-variance functions for residuals between traits within environment are also needed.

Note that estimates of reaction norm models for residual effects are not needed to model genetic gain in a target environment based on data collected in the environments for which character-state parameter estimates are available, i.e. the nucleus and challenge environment in the example. However, when evaluating phenotype recording in alternate environments, reaction norm estimates of the residual variance–covariance structure are required.

In the example, unobserved environmental correlations were set equal to their corresponding genetic correlations. If individuals are phenotyped in only one environment, only environmental correlations among traits within an environment matter and impact predictions of response to selection, including response in the target environment. However, the values assumed for the unobserved environmental correlations (i.e. between traits in the nucleus and the challenge environment) do affect the environmental reaction norm parameters and, therefore, environmental correlations computed in another recording environment. Thus, assumptions made about unobserved environmental correlations could affect optimization of the recording environment based on the reaction norm models. The impact of this is, however, expected to be limited.

In all analyses, parameter estimates (heritabilities and genetic and environmental correlations, as well as the environmental covariates) were assumed to be known without error. The impact of errors in parameter estimates needs further evaluation. The impact of errors in parameters of the character-state model will be exacerbated for estimates of parameters in unobserved environments through the reaction norm models, in particular for environments that are outside the range of observed environments.

## Conclusions

Equivalencies between reaction norm and character-state models for multiple-trait phenotypes were successfully derived at both the genetic and environmental level. These equivalencies allow multiple-trait reaction norm parameters to be derived from multiple-trait character state parameters, and vice versa. The developed methods can be used to optimize genetic improvement programs for a multiple-trait breeding goal in a target environment without recording phenotypes and estimating parameters for the target environment.

## Data Availability

Not applicable.

## References

[1] Falconer D (1993). Quantitative genetics in Edinburgh: 1947–1980. Genetics.

[2] Schaeffer LR, Dekkers JCM. Random regressions in animal models for test-day production in dairy cattle. In: Proceedings of the 5th World Congress on genetics applied to livestock production: 7–12 August 1994; Guelph; 1994.

[3] de Jong G, Bijma P (2002). Selection and phenotypic plasticity in evolutionary biology and animal breeding. Livest Prod Sci.

[4] Kolmodin R, Bijma P (2004). Response to mass selection when the genotype by environment interaction is modelled as a linear reaction norm. Genet Sel Evol.

[5] Van Tienderen PH, Koelewijn HP (1994). Selection on reaction norms, genetic correlations and constraints. Genet Res.

[6] Henderson CR (1982). Analysis of covariance in the mixed model: higher-level, nonhomogeneous, and random regressions. Biometrics.

[7] Sae-Lim P, Mulder H, Gjerde B, Koskinen H, Lillehammer M, Kause A (2015). Genetics of growth reaction norms in farmed rainbow trout. PLoS One.

[8] Knap PW, LiZhen W. Robustness in pigs and what we can learn from other species. In: Proceedings of the 8th World Congress on genetics applied to livestock production: 13–18 August, 2006; Belo Horizonte; 2006.

[9] Wei M, Van der Steen H (1991). Comparison of reciprocal recurrent selection with pure-line selection systems in animal breeding (a review). Anim Breed Abstr.

[10] Wientjes Y, Calus M (2017). Board invited review: the purebred-crossbred correlation in pigs: a review of theory, estimates, and implications. J Anim Sci.

[11] Putz AM, Harding J, Dyck MK, Fortin F, Plastow GS, Dekkers J (2019). Novel resilience phenotypes using feed intake data from a natural disease challenge model in wean-to-finish pigs. Front Genet.

[12] Mulder HA (2016). Genomic selection improves response to selection in resilience by exploiting genotype by environment interactions. Front Genet.

[13] Rutten MJM (2002). SelAction: software to predict selection response and rate of inbreeding in livestock breeding programs. J Hered.

[14] Cheng J, Putz AM, Harding JCS, Dyck MK, Fortin F, Plastow GS (2020). Genetic analysis of disease resilience in wean-to-finish pigs from a natural disease challenge model. J Anim Sci.

[15] Wray NR, Hill WG (1989). Asymptotic rates of response from index selection. Anim Sci Prod.

[16] Gienapp P (2018). The choice of the environmental covariate affects the power to detect variation in reaction norm slopes. BioRxiv.

[17] Fikse W, Rekaya R, Weigel K (2003). Assessment of environmental descriptors for studying genotype by environment interaction. Livest Prod Sci.

[18] Yates F, CocHRAN WG (1938). The analysis of groups of experiments. J Agric Sci.

[19] Finlay K, Wilkinson G (1963). The analysis of adaptation in a plant-breeding programme. Aust J Agric Res.

[20] Guy SZY, Li L, Thomson PC, Hermesch S (2019). Reaction norm analysis of pig growth using environmental descriptors based on alternative traits. J Anim Breed Genet.

[21] Kolmodin R, Strandberg E, Madsen P, Jensen J, Jorjani H (2002). Genotype by environment interaction in Nordic dairy cattle studied using reaction norms. Acta Agric Scand A Anim Sci.

[22] Su G, Madsen P, Lund M, Sorensen D, Korsgaard I, Jensen J (2006). Bayesian analysis of the linear reaction norm model with unknown covariates. J Anim Sci.

[23] Carvalheiro R, Costilla R, Neves HH, Albuquerque LG, Moore S, Hayes BJ (2019). Unraveling genetic sensitivity of beef cattle to environmental variation under tropical conditions. Genet Sel Evol.

[24] Pereira RJ, Ayres DR, Faro LE, Filho AEV, Verneque RdS, Albuquerque LGd (2013). Genetic parameters for production traits of dairy Gyr (*Bos indicus*)×Holstein cattle estimated with a random regression model. Livest Sci.

[25] Chiaia H, De Lemos M, Venturini G, Aboujaoude C, Berton M, Feitosa F (2015). Genotype × environment interaction for age at first calving, scrotal circumference, and yearling weight in Nellore cattle using reaction norms in multitrait random regression models. J Anim Sci.

[26] Meyer K (2001). Estimating genetic covariance functions assuming a parametric correlation structure for environmental effects. Genet Sel Evol.

[27] Schnyder U, Hofer A, Labroue F, Künzi N (2001). Genetic parameters of a random regression model for daily feed intake of performance tested French Landrace and Large White growing pigs. Genet Sel Evol.

[28] Kirkpatrick M, Heckman N (1989). A quantitative genetic model for growth, shape, reaction norms, and other infinite-dimensional characters. J Math Biol.

[29] Kirkpatrick M, Lofsvold D, Bulmer M (1990). Analysis of the inheritance, selection and evolution of growth trajectories. Genetics.

